# Supplementary material for: The Clinical Feasibility and Safety of 1.5 T MR-Guided Daily Adapted Radiotherapy in 1000 Patients: A Real-World Large Experience of an Early-Adopter Center
Source: Cancers (Basel). 2025 Jun 17;17(12):2012. doi: 10.3390/cancers17122012 (PMC12191406; doi:10.3390/cancers17122012)
Supplement: Supplementary file 1 [file cancers-17-02012-s001.zip › cancers-3608694-supplementary.pdf]

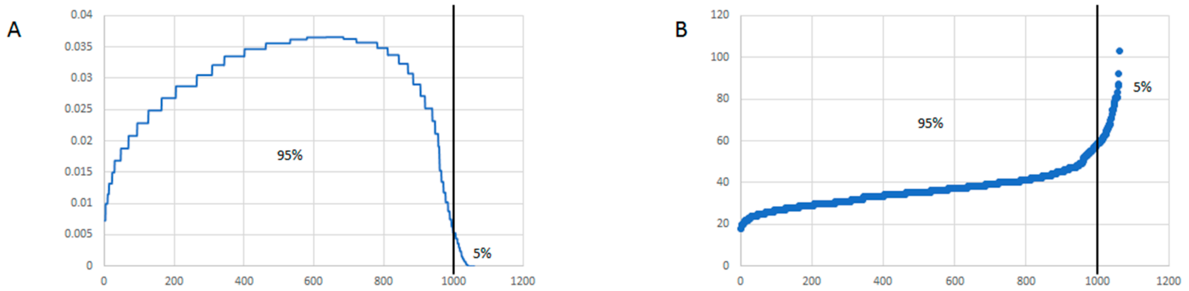

Figure S1. Normal distribution (A) and scatter plot distribution (B) of the total of 1061 treatment time measurements. 5% of the values at the extreme right tail of the curve could be considered outliers, explanation in the main text.

Table S1. First and second most applied fractionations according to RT site.

RT site	RT dose/ fraction schedules	percentage
prostate	36.25 Gy in 5 fractions	53.6%
	35 Gy in 5 fractions	30.5%
prostate bed	67.5 Gy in 30 fractions	35.2%
	30 Gy in 5 fractions	33.3%
prostate/ prostate bed + pelvic lymphnodes	67.5 Gy in 30 fractions	42%
	60 Gy in 20 fractions	38%
lypmhnodes	35 Gy in 5 fractions	51.2%
	30 Gy in 5 fractions	14.2%
pancreas	45 Gy in 6 fractions	30%
	42 Gy in 6 fractions	15%
liver	50 Gy in 5 fractions	25%
	45 Gy in 5 fractions	16.7%

Table S2. Number of treatment courses and average in-room time per fraction per year (n=1061).

Treatment time period	Treatment courses	Average in-room time
10/2019-09/2020	170	44 minutes
10/2020-09/2021	254	42 minutes
10/2021-09/2022	220	34 minutes
10/2022-09/2023	238	35 minutes
10/2023-06/2024	179	34 minutes

Table S3. Average treatment time according to RT site (n=1061).

RT site	Average in-room time
liver	44 minutes
pancreas	42 minutes
arenal gland	40 minutes
prostate/ prostate bed +/- pelvic lymphnodes	38 minutes
lung	37 minutes
lymphnodes	36 minutes
bone metastases	32 minutes
cerebral tumours	28 minutes
others	35 minutes
